# Getting under the skin of the primary care consultation using video stimulated recall: a systematic review

**DOI:** 10.1186/1471-2288-14-101

**Published:** 2014-08-30

**Authors:** Zoe Paskins, Gretl McHugh, Andrew B Hassell

**Affiliations:** 1 Arthritis Research UK Primary Care Centre, Primary Care Sciences, Keele University, Keele, Staffordshire ST5 5BG, UK; 2 School of Nursing, Midwifery & Social Work, University of Manchester, Oxford Road, Manchester M13 9PL, UK; 3 School of Medicine, Keele University, Keele, Staffordshire ST5 5BG, UK

**Keywords:** Doctor-patient consultation, Primary care, Video, Recall

## Abstract

**Background:**

Video stimulated recall (VSR) is a method of enhancing participants’ accounts of the consultation using a video recording of the event to encourage and prompt recall in a post consultation interview. VSR is used in education and education research, and to a lesser extent in medical and nursing research. Little is known about the sort of research questions that lend themselves best to the use of VSR or the impact of the specific VSR procedure on study quality. This systematic review describes studies in primary care that have used the method and aims to identify the strengths, weaknesses and role of VSR.

**Methods:**

A systematic literature search has been conducted to identify primary care consultation research using VSR. Two authors undertook data extraction and quality appraisal of identified papers and a narrative synthesis has been conducted to draw together the findings. In addition, theory on classifying VSR procedures derived from other disciplines is used as a lens through which to assess the relevance of VSR technique.

**Results:**

Twenty eight publications were identified that reported VSR in primary care doctor-patient consultation research. VSR was identified as a useful method to explore specific events within the consultation, mundane or routine occurrences, non-spoken events and appears to particularly add value to doctor’s post consultation accounts. However, studies frequently had insufficient description of methods to properly evaluate both the quality of the study, and the influence of VSR technique on findings.

**Conclusions:**

VSR is particularly useful for study of specific consultation events when a ‘within case’ approach is used in analysis, comparing and contrasting findings from the consultation and post-consultation interview. Alignment of the choice of VSR procedure and sampling to the study research question was established as particularly important in the quality of studies. Future researchers may consider the role of process evaluation to understand further the impact of research design on data yielded and the acceptability of the method to participants.

## Background

The consultation has been long a subject of interest for researchers seeking to gain further understanding of the doctor-patient relationship and interaction. In 1969, Byrne and Long audio recorded over 2500 consultations to research verbal behaviours between doctors and patients [1]; since then, there has been increasing use of video recordings to facilitate observational consultation research [2]. An alternative method to indirect observation of the consultation is to seek participants’ accounts of events by interview, focus group or workshops and these methods have been used in a recent publication by The Health Foundation ‘When doctors and patients talk: making sense of the consultation’ [3].

Participant accounts are retrospective and limited to that which is remembered and reported; however, recall accuracy and completeness may be enhanced by playing back the video-recorded consultation within the interview context: ‘video stimulated recall’ (VSR). VSR may be useful for improving recall, for uncovering cognitive processes and as a tool to facilitate reflections on elements of many different social interactions. The method of VSR has been used extensively in educational and counselling research [4] and to a lesser extent in medicine and nursing. When data derived from participant accounts using VSR is combined with consultation analysis an in-depth exploration of consultation events may be achieved.

Stimulated recall can also be achieved with the use of audio recordings, in place of video. However, the advantage of using video recordings is that the visual stimulus may be a stronger stimulus for recall and the participant may also comment or reflect on their non-verbal behaviours.

VSR is described as useful for the study of patient-professional interactional components of the consultation and complex, context dependent occurrences, in addition to permitting more accurate recall of events that may have been forgotten [5]. The technique is also complex, costly and time consuming and it is suggested it should be reserved for research questions that cannot be answered with consultation analysis or participant interviews alone [5,6]. Henry at al [5] conducted a literature review of studies using the method; however in the absence of quality appraisal of the studies, no empirical evidence was presented to guide future researchers in the most appropriate use of VSR or to illuminate the methodological strengths and weaknesses particular to VSR. The question remains as to which types of research question lend themselves best to this method.

VSR may be conducted in a number of different ways. For example, the video may be shown in entirety prior to a semi-structured interview or the participant may be asked to comment during playback on specific areas of interest. The nuances of VSR procedure are considered important in the design of research although they have not been previously described in medical literature.

In summary, VSR appears to be an important methodology for researching the consultation but what is missing from the existing literature is an understanding of the strengths and weaknesses particular to the method, the way in which VSR procedure relates to study quality and the research questions that may be best suited to the method. This systematic review aims to address these gaps, and was conducted as preliminary work for a study that planned to use the method of VSR in exploring the content of osteoarthritis consultations in primary care. In this systematic review, we describe studies in primary care consultation research that have used VSR, in order to describe the utility of the method in consultation research.

### Specific objectives of this review

This systematic review aims to further understanding of the role of VSR in doctor-patient consultation research to describe:

**Table 1:** **Components of SR procedure and theoretical effect on outcome (adapted from Gass and Mackey **[7]**)**

	**Example/comments**
**Time between video recorded event and SR**	Participant recall of events will be greater immediately after the interview.
**Strength of stimulus**	Video is an example of a strong stimulus, but the strength of stimulus may be increased still further by additional stimulus for recall e.g. transcripts of consultation. The greater strength of stimulus, the more enhanced the recall will be.
**Procedural structure of accompanying interview**	A structured interview is an example of high procedural structure and will result in more specific information relative to the research question.
	A low structure approach would involve minimal questioning and the use only of neutral prompts during playback e.g. “what were you thinking then?”. This method may be more suitable where the research question concerns cognitive processes at the time of the interview and is less likely to result in researcher contamination.
**Initiation of recall event**	The researcher may lead recall by asking the participant to comment on areas of interest to the researcher, or the participant may be asked to comment on aspects of their choice. Again, researcher initiated events may encourage more reflection than recall alone.
**Relationship between video recorded event and line of inquiry**	During a VSR interview, a participant may be questioned only on events that occurred during the video, described as a ‘concrete relationship to action’. However, they may be asked to abstract to other general events, an example of a ‘non-specific relationship to action’. In this instance, their recall may not be as great.
**Participant training**	Participants may need training and practice if asked to comment on stimulus in an unstructured way. Training may enhance a participant’s ability to reflect on observed events.

a. The research questions that have been addressed using VSR

b. The methodological strengths and weaknesses particular to VSR, including its acceptability to participants

c. The procedure of VSR (using the theoretical framework in Table 1) and how the choice of procedure influences overall considerations of study quality and utility

d. The areas of research where VSR adds value

## Methods

### Theoretical framework: VSR procedures

Gass and Mackey [7] have previously described a classification of SR techniques; this was used as a theoretical framework to inform analysis. One inherent limitation of the technique of VSR is that the feelings and thoughts expressed in the context of a post-consultation interview may not reflect the thoughts at the time of the consultation, and are subject to researcher influence [4,5]. Careful attention to the procedure of VSR may reduce this effect. Techniques of VSR vary widely and different methods may be more suited to capturing recall, reliving or reflection [5]. Gass and Mackey have reviewed the literature across different disciplines in the techniques of stimulated recall (SR), although not restricted to video, in their text relating to second language research [7]. Their methodological theoretical framework has applications beyond language research and is considered a useful starting point for researchers considering the method [4]. In Table 1, the techniques of SR are listed, as adapted from Gass and Mackey’s classification [7].

In theory, the recall accuracy will be greatest if the interview takes place immediately after the consultation event, with the highest strength of stimulus and if the stimulus has a concrete relation to the area of questioning. Concern is reported in the psychology literature about the types of memory accessed with delayed recall which is reported to affect validity of responses. However, as short term memory decays within a few hours, it is suggested that there may be not much difference in recall performed at 3 hours, compared with 3 days [7].

Lyle [4] argues research questions concerned with decision making or cognitive processes *during* the video recorded event (in this case, the consultation) are most likely to be subject to reinterpretation of proceedings; for this reason, the choice of structure of the post consultation interview and the individual initiating recall are key to reduce the likelihood of reflection. The wording of questions would therefore seem to be of great importance in reducing researcher contamination. There is some empirical evidence for this from a number of studies in psychology around ‘think aloud’ protocols. Although these do not strictly represent SR, a participant is asked to verbalise thoughts while completing a task. Ericsson and Simon have conducted many reviews on this subject and their consistent finding is that verbalisation during a task does not change performance *unless* participants are asked to verbalise motives or reasons for their behaviour; in this case, participants are observed to change behaviour. This finding is attributed to participants speculating or theorising about higher cognitive processes that may be automatic [8].

### Literature search

Based on the assumption that in primary care the consultation may differ in character and structure from secondary care settings, this review is restricted to studies in primary care. The search was divided into four areas: consultation; primary care; video; and qualitative research. The literature search was conducted in March 2012 and repeated in November 2012 in Medline, Psychinfo, CINAHL, Embase and HMIC, Web of Science and BIOSIS. Additional references were obtained by reference checking, contacting experts, searching conference abstracts and cited reference checking using Web of Science. The search was limited to English language publications.

Given the wide range of terms used for video-elicitation and the possibility that terms exist of which the authors of this review are unaware, the search was left broad and all results relating to video searched for details of stimulated recall. If a post consultation interview was reported in the abstract the full text was reviewed to establish if VSR had been used.

A full list of search terms appears in Table 2 and the full Medline Search in Additional file 1. Inclusion and exclusion criteria are listed in Table 3.

**Table 2:** Search terms used

**Consultation**	**Primary care**	**Video**	**Qualitative research**
Consultation	Primary health care	Video	Qualitative
Communication	Family medicine	Film	Experience
Doctor (or physician, clinician) patient relationship (or talk or rapport or relations)	Family practice	Recording	Attitudes
	General practice	Videodisc	Findings
	GP	Videotape	Interviews
	Family physicians	Digital recording	Theme
	Family doctor		Account

Note: terms within columns combined with OR operator, results across columns combined with AND operator.

**Table 3:** Inclusion and exclusion criteria

**Inclusion criteria**	**Exclusion criteria**
Studies in primary care	Hospital-based studies, including outpatient clinics
Observational studies of “real life” GP-patient consultations	Papers written in languages other than English
Studies that have used video to record the consultation	Video-recorded consultations not shown to research participants
Studies that have showed the video-recorded consultation to research participants as part of further data collection	Educational research studies concerned with making assessment of doctor or trainee performance
Describes research question and results, not just methodology	Consultation with other healthcare practitioner (e.g. nurse, physiotherapist)
	Experimental studies or trials
	Studies involving children
	Studies using actors or standardised patients

In the first stage of sorting all record titles were screened and exclusions made where possible by the first author (ZP). The remaining records were then viewed as abstracts, by two reviewers independently (ZP and GMcH), and exclusions made where possible. Those titles and abstracts not fulfilling the inclusion criteria at each stage were discounted. The full text of the remaining articles was then requested, including those with no abstracts. Disagreements between the reviewers were resolved by discussion and consensus on inclusion or exclusion reached for both abstract and full text review.

All full text articles retrieved were read, decisions made regarding their inclusion, and the reasons for exclusion recorded, again by two reviewers. Exclusions were made serially by each criteria and only one reason recorded for each abstract or full text discounted. An access database containing the data extraction and quality assessment items was designed and piloted by two reviewers and minor amendments made. Thereafter, data extraction and quality appraisal forms were completed for each paper by two reviewers (ZP, and either GMcH or AH) independently. Two papers described methodology only [6,9], with no independent research question; these were not counted in the final sample, but the content of each used to aid quality appraisal of their respective related paper.

### Quality assessment

A list of characteristics for quality assessment was designed, based on the following two sources:

**Table 4:** Quality assessment items derived from CASP checklist

1.	Was the research design appropriate to address the aims of the research?
2.	Was the recruitment strategy appropriate to the aims of the research?
3.	Were the data collected in a way that addressed the research issue? [This was adapted to 2 sub questions ‘was the data collection clearly described’ (as without this it is not possible to answer whether data collection is appropriate or not) and ‘was the data collected in an appropriate way to address the research question?’]
4.	Has the relationship between researcher and participants been adequately considered?
5.	Have ethical issues been taken into consideration?
6.	Was the data analysis sufficiently rigorous? This includes whether the analysis process is clearly described
7.	Is there a clear statement of findings?
8.	How valuable is the research?
	[this has been incorporated into the ‘Reviewer’s main conclusions’ – see Table 5, and Additional file 2]

1. Coleman [2] cites four aspects of ‘bias’ of research using video, namely the effect of the video-recorder on the patient and GP (described as internal validity) and the characteristics of patients and GPs who consent to being videotaped, compared with non-consenters (described as external validity). The extent to which authors reported on these aspects was recorded.

2. Papers included used qualitative methodology as a framework for analysis and so questions from the Critical Appraisal Skills Programme (CASP) Qualitative appraisal tool [10] were incorporated in the checklist. This tool has been used in other qualitative systematic reviews [11]; the eight detailed questions from CASP included are detailed in Table 4.

### Data extraction

The data extraction elements are shown in Table 5. The full data extraction form used by the authors, including the quality assessment is included in Additional file 2.

**Table 5:** Data extraction questions

1.	What is the research question?
2.	How were consultations selected?
3.	Who were the population of interest?
4.	How many consultations were videotaped? How many were analysed?
5.	What methods have been used for analysis of the consultation?
6.	Has the visual data been analysed?
7.	Who was subsequently shown the videotapes? (patient or GP)
8.	How many interviews were conducted?
9.	How were the videotapes in the interviews selected?
10.	What format did the interview take? (i.e. how the video playback was incorporated in the interview)
11.	What was the analysis method of the interviews?
12.	Has the researcher commented on the acceptability of the research method to participants?
13.	To what extent did each element of data collection contribute to the findings?
14.	What are the main findings?
15.	What are the authors’ main conclusions?
16.	What are the reviewer’s main conclusions?
17.	Did each component (interview vs video) contribute to the findings?
18.	To what extent did the VSR interview add to the research findings?

### Synthesis

A narrative synthesis approach was used, guided by the aims of the review; this method is ideally suited to combining results from qualitative studies where quantitative synthesis is not possible and easily adaptable to describing process (methods) rather than pooling study results [12]. The outline of SR techniques described by Gass and Mackey [7] was used as a theoretical framework to inform analysis. Following individual data extraction and quality appraisal, authors met to first discuss and compare findings for each study. Secondly, emergent patterns and themes across studies were discussed. Thereafter, a preliminary synthesis was achieved using tabulation of studies and forming groups and moderator variables used to explore relationships between studies. All authors then contributed to the final report. The study methods and the reporting of results adhere to the guidelines in the PRISMA statement.

## Results and discussion

### Identification of studies

**Figure 1:**
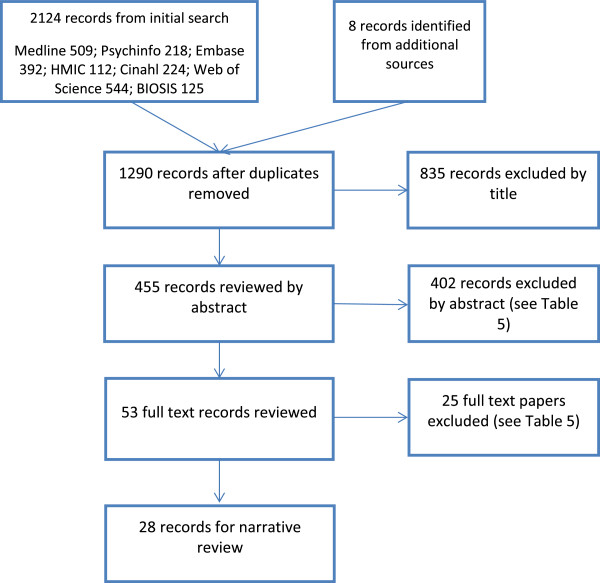
Phases of identification of papers.

2132 papers were identified by the initial search, and 28 ultimately fulfilled inclusion criteria. A flowchart showing the phases of identification, as recommended by PRISMA, is shown in Figure 1, and Table 6 details the reasons for exclusion.

**Table 6:** Reasons for exclusion

**Reason for exclusion**	**Number excluded from abstracts**	**Number excluded from full text**
Setting: not primary care	14	0
Participants: GPs not included	15	0
Method: did not include video recorded consultations	198	9
Method: Consultations not ‘real life’	42	1
Method: video not shown to research participants	133	12
Described method only, no research questions or results	0	1
Full text unavailable	N/A	2
Total	402	25

### Description of included studies

The included articles are described in terms of research question and area in Table 7. The 28 individual articles refer to 18 sets of video recorded consultation data, and multiple publications from a single data set are listed together in a single row. The areas of research fall into eight categories: decision making; communication (including a subset of cross-cultural communication); doctor-patient relationship; patient experiences; evaluation of the method of VSR; self-management; health promotion and team working. Many of the studies were concerned with generic aspects of the consultation and as such have a relatively unselected sample. However, five studies were focused on specific consultation content: patients’ expressed psychological problems [13]; discussion of smoking cessation [14]; HIV risk [15]; self-management in long term conditions [16] and health promotion [17]. In justifying the choice of method, many sought simply to gain a fuller understanding of participants’ experiences. The doctor-patient relationship and communication were the most common areas of inquiry with three studies researching the effect of the computer on the relationship, and three looking specifically at cross-cultural communication. Specific events within the consultation were the focus of the study in studies concerning decision-making, or discussions around HIV risk and smoking cessation. Two studies used the method to explore non-deliberate behaviour: unspoken information or non-verbal cues [18,19].

**Table 7:** Description of included studies

**First author and year**	**Research question**	**Population/consultations of interest**	**Area of research**
Ali [20]	To provide a detailed understanding of the ways in which white and South Asian patients communicate with white GPs and to explore any similarities and differences in communication	South Asian patients	Communication: cross cultural
Als [21]	To identify patterns of GP and patient behaviour related to computer and to identify patient and doctor perceptions of the computer	Unselected	Doctor patient relationship: Impact of computer
Arborelius [17,22-28]	To describe and evaluate a stimulated recall methodology	Unselected (but stratified with respect to age & gender)	Evaluation of VSR method
	To study the difficulties and dilemmas a GP faces during daily consultations		‘Difficult’ consultations
	To understand phenomena in consultations where the GP has expressed difficulties		Doctor patient relationship
	To compare the patients' and the doctors' comments on video-recorded consultations in order to increase understanding of shortcomings in patient-doctor relationship		
	To describe and understand the experiences of general practitioners in consultations		
	To describe and understand patients' positive and negative experiences of General Practitioners		
	To describe the specific behaviour in consultations where the patient experiences a satisfying human relationship with the GP		
	To characterize health counselling discussion in the consultation	Subsample where health promotion discussed	Health promotion advice
Blakeman [16,29]	To explore self-management support in primary care consultations	Patients with long term conditions	Self-management
	To explore the relevance of computer information systems in self-management dialogue		Impact of computer
Bugge [19]	To investigate incidences, consequences and reasons for non-disclosure of information in decision making	Consultations in family planning clinic and diabetes clinic^a^	Decision making
Cegala [30]	To compare doctor and patient views on communication during the consultation	New and follow up patients	Doctor-patient relationship
Coleman [14]	To elicit, relate and interpret GP accounts of why they discuss smoking with some patients and not others	Patients who smoke	Decision making
Cromarty [31]	To describe the range and type of thoughts patients have during their consultations	Unselected	Patients experiences
Epstein [15]	To describe the structure of HIV related discussion, characterise effective and efficient communication and identify common difficulties	Consultations where HIV risk is discussed	Communication
			Doctor-patient relationship
			Difficult consultations
Frankel [32]	To understand the characteristics of the ‘optimal healing environment’ in the consultation	Established patients presenting to doctors with a range of satisfaction scores	Doctor- patient relationship
Gao [33]	To explore the influence of cultural practices on discussion of colorectal screening	Patients having colorectal screening recommendations	Communication: Cross cultural
Henry [18]	To understand the impact of tacit clues on making judgements in the consultation	Patients undergoing health maintenance examinations	Decision making
Rosenburg [34,35]	To understand what occurs in a triadic encounter	Triadic Consultations involving an interpreter	Communication
	To delineate differences in encounters between professional and family interpreters		
Rosenburg [13]	To explore the communication patterns and perceptions between family doctors and psychologically distressed immigrant patients?	Immigrant patients with psychological problems	Communication: Cross cultural
Saba [36]	To examine shared decision making and the experience of partnership of the doctors and patients	Stratified sample of patients presenting with diabetes or hypertension	Shared decision making
Timpka [37]	To compare the experiences of patients and care givers of consulting across the primary care team	Patients who encountered more than one team member in a visit	Clinician-patient relationship and team working
Treichler [38]	To identify and explore the power relations in a triadic consultation with GP, patients and medical student	Traidic consultation with medical student	Doctor-patient relationship
Ventres [39,40]	To explore how electronic health record affects encounters between physicians and patients	Unselected	Doctor-patient relationship: impact of computer

^a^And other non-primary care consultations.

### General methodological considerations

The results from the quality appraisal are detailed in Table 8. Frequently, studies had insufficient detail in their methods section to properly evaluate the quality of the study. Three author groups described their methodology in separate publications [5,6,9,22]. Some authors also reported analysis of different data components in separate publications where there were individual research questions [9,14,16,17,20,22-27,29,39-42]. These associated publications were not always referenced in the included study [20,42]. Multiple publications on the same dataset were generally not felt to be of high methodological quality, predominately due to the lack of alignment between research question and methods, particularly participant sampling. For example, Arborelius et al. [17] focused one paper on health promotion advice when only 8 of the original 46 video recorded consultations contained discussion of this nature.

**Table 8:** Findings from quality appraisal

**First author and year**	**Sampling and consent**	**Effect of video or study methods on behaviour**	**Other methodological issues identified from QA using CASP tool**
Ali [20]	No mention.	States GPs were recorded over a period of time to try and reduce effect	Mentions inclusion criteria but doesn’t describe these. Not clear in interview if interpreter was used or not, and what questions the patient was asked. Analysis not clearly described. Conclusions appear to be derived from literature review rather than empirical findings.
	Characteristics of consenters described in unreferenced related paper only		
Als [21]	States attempted to recruit a sample of variation, characteristics and consent not described	No mention	Analysis not described in detail.
Arborelius [17,22-28],	Characteristics of consenting patients described but not non-consenters.	Mentions in 2 papers the influence of the camera was minimal (self-report from participants)	Participant comments during VSR often not aligned to research question as only neutral prompts, therefore small number of comments relevant to study aims [23,24].
	Research question not aligned to sampling resulting in small numbers of relevant consultations for some papers [17,25].		Analysis clearly described in 2 papers in this group [23,27].
			Possible over-interpretation of participants’ comments (particularly assumptions on when GP had failed to ‘grasp’ situation) [25,27] with limited discussion of implication of findings [24]
			Analysis mostly conducted across case and not within case: within cases analysis and comparison may have enhanced analysis and understanding of cases where difficulties exist in the consultation [26] (where within case approach was used, only 1 minute of consultation analysed [23]).
Blakeman [16,29]	Characteristics of consenting patients and GPs described but not non-consenters.	No mention	Data collection, rationale for study and analysis described in detail. Possible limited conclusions to be drawn from the study of one consultation when studying self-management support which may happen longitudinally in the doctor patient relationship.
			Only empirical quotes from nurses reported in 2nd paper, yet conclusions refer to doctors and nurses. In 2nd paper, no discussion about how context of nurse or doctor consultation would influence findings in relation to QOF.
Bugge [19]	Characteristics of consenting patients described but not non-consenters. Limited characteristics of GPs described	Brief mention as limitation	Relative contribution of different post consultation interviews not described (3 per participant).
			Analysis well described.
Cegala [30]	Characteristics of consenting patients and GPs described but not non-consenters.	No mention. Effect on behaviour may be more likely as consultation taken out of normal surgery context and separate microphone on table.	Paper based on assumption that participant’s spontaneous comments during playback (with no guided prompts) can be used to draw conclusions about patient perceptions of doctor competence in communication exchange.
	No information about sampling.		No empirical quotes to support findings.
Coleman [14]	Characteristics of consenters and non-consenters presented. GPs sampled to represent a range of attitudes to smoking	Discussed as potential limitation.	Quantitative methods to support sampling helped gain a maximum variation sample.
			Analysis well described.
			Author’s role as GP and peer to GP participant’s not explored.
Cromarty [31]	No mention of details of video selection or recruitment (videos selected by participating GPs and not researcher)	No mention	Relative contribution of different phases of post consultation interview not described (unprompted, with video recall and then written transcript).
			Analysis not described in depth.
Epstein [15]	Characteristics of consenting patients and GPs described but not non-consenters.	One comment that GPs stated not affected.	Robust analysis strengthened by different approaches including coding of behaviours, attention to conversation flow and classification scheme of the level and depth of discussion of HIV risk.
	Discussion of how GPs volunteering to be video recorded may not be representative of GP population.		More than one consultation per GP facilitated robust analysis.
	Purposive sampling used to identify patients/ consultations more likely to contain discussion of HIV risk		Not clear how video shown or VSR procedure.
Frankel [32]	No mention	No mention	Research question or theoretical framework lacking.
	Sample size unclear		Participant comments (GP or patient) on video not confidential and revealed to other participant. Consent not mentioned.
Gao [33]	Characteristics of consenting patients described but not non-consenters. Limited characteristics of GPs described	No mention	Recruitment strategy not entirely appropriate: GP interviews not needed to answer research question and weren’t utilised.
			Three stage analysis clearly described.
Henry [18]	Variation sampling of patients to gain mix of gender, age and race. GPs sampled with respect to years in practice and specialty	No mention	Insufficient detail about structure of interview or VSR procedure to judge how appropriate study method was for exploring tacit clues.
			No discussion of how context of health maintenance consultations might influence findings.
Rosenburg [34,35]	Characteristics of sample described (patients and interpreters), but not non-consenters	No mention	Conclusion not supported by results and patient views would have added value and been relevant to research question [34].
			Little information about VSR procedure of format of interview [35].
Rosenburg [13]	Recruitment well described. Characteristics of sample described, but unclear how many underwent VSR	No mention	Method successful in identifying consultations of interest and evidence supports authors’ conclusions. No discussions of limitations.
			Patients made few comments over video and structure of interview not clear.
Saba [36]	Characteristics of sample described but low consent rate not discussed.	Brief mention of possible effect	Robust analysis strengthened by different approaches including analysis within and across cases, contrasting observed and subjective experiences of shared decision making to construct typology of SFM archetypes and using themes from interviews.
Timpka [37]	Characteristics of consenting patients described but not non-consenters.	Brief mention of possible effect	Complex study but not clear how much video the participants viewed, the instructions the participants were given when watching the video or the consent arrangements.
			Conclusion not supported by results.
Treichler [38]	Case study of one patient. No mention of sampling.	No mention	Limitations associated with the study of one consultation.
Ventres [39,40]	Not described	Brief mention	Analysis well described but no empirical quotes to support findings. More description of consultation context would have increased credibility of findings.

Sampling emerged as a particularly important component of quality in research design. For example, Coleman et al. ensured richness of data in their video data about smoking cessation by sampling at every stage of the method; GPs were sampled to represent a range of attitudes to smoking identified on a questionnaire, patients were selected on basis of smoking status and the videos shown to the GPs were chosen to reflect a range of different types of discussion around smoking e.g. smoking cessation discussed in the presence or absence of smoking related problem [14]. Epstein et al. also enhanced sampling by using pre-consultation questionnaires to identify patients for their sample concerned about HIV risk. Although a number of studies described the characteristics of the sample of their study, only one did this with reference to non-consenters enabling the reader to judge the transferability of the results [14].

Five studies only analysed VSR data from either patient or doctor, 10 used VSR data from more than one perspective (patient, doctor or interpreter) and 13 studies analysed both VSR and consultation data together. The research question did not always match the data collected; for example in four studies researching communication [13,20,34,35], the VSR interviews were the only data analysed and analysis of the consultation itself may have added value. Furthermore, three of these studies did not study all parties in the consultation.

Conversely, in two studies, the study findings did not appear to represent all the different data sources collected. Gao et al. [33] researched communication, looking in detail at cross-cultural influences on colorectal screening; in their study only patient VSR and consultation findings are reported despite the methods indicating they also conducted VSR with GPs. Blakeman et al. [29] interviewed both doctors and nurses in their study regarding the influence of the Quality and Outcomes Framework (QOF). The doctor responses appeared to be underrepresented in the results; in this instance this may have been due to the context of the study as nurse consultations may have been more QOF orientated.

In terms of the effect of the video on participants’ behaviour, two studies reported that GP behaviour was not affected by the video [15,40]. Arborelius et al. [22] asked GPs if they thought their behaviour was altered on a questionnaire pre and post viewing; 80% reported feeling slightly or not affected, which increased to 90% post viewing of the video. The physicians felt more affected by the presence of the camera than patients. Four other studies mention this as a limitation with no studies giving any empirical evidence to support or refute an effect.

Most studies limited their discussion about ethical implications of the study to a statement about ethics board approval (10 datasets) or that participants consented (14 data sets). In one study, patients were video recorded before their consent was given [37]. Due to the brevity or absence of statements about ethical issues, it was usually unclear what participants had been told was the purpose of the study. In studies where doctor deficiencies were the clear focus of the paper, one wonders if participating GPs knew this in advance, and whether they would have agreed to participate if they had known. In one exception to this, Coleman et al. [14] state that GPs did not know the study was about smoking, presumably to reduce influence of the study on the behaviours and talk of interest. A few studies referred to anonymity and confidentiality, and gave participants the option to withdraw [22,26]. Epstein et al. [15] disclosed that some GPs were ‘visibly upset’ when viewing the videos.

The influence of the researcher on the research process was generally under-recognised. Indirectly, this was alluded to in studies using neutral prompts during video playback and participant led recall, to reduce researcher influence. However, beyond this there were no critical reflections whereby authors considered their own role in the research process.

### Acceptability to participants

No studies directly addressed the issue of acceptability of the method to participants. Patient participants have expressed the novelty of watching themselves on screen and directed a number of their comments during playback around this issue. In one dataset, the authors purposely showed the video first in an introductory manner so that participants could become more used to watching themselves on screen, noting that patients ‘comment in a neutral and polite way’ [17,22-27]. Acceptability of the method can be inferred to some extent by participant consent rates but only 6 datasets recorded consent rates of patients in any associated paper and none indicated consent rates of GPs. Interestingly, Blakeman et al. [43] did not incorporate patient VSR into their study design as they anticipated this would be unacceptable to participating GPs. Blakeman has since indicated this assumption was probably unfounded (personal communication).

### VSR Procedure: relationship to research question and study quality

In the Introduction, a classification of six elements of VSR procedure was introduced (Table 1). This classification comprises: time interval between consultation and VSR; strength of stimulus; structure of interview; who initiates recall; relationship between line of questioning and stimulus and participant training. This classification was used as a lens through which to view the included studies in this review. Table 9 details the procedures used in each study using this classification. Participant training was not described in any study and similarly the relationship of events on the video to the researchers’ line of inquiry in interview was difficult to evaluate in the absence of an interview schedule and so these two elements are not included in the Table.

**Table 9:** Techniques of VSR compared with area of research and data used for triangulation

**Ref**	**Area of research**	**Sample size**^**a**^	**Interval between consultation and interview**	**Nature of stimulus**	**Initiation of recall**	**Procedural structure**	**Data used in analysis**
					**Participant (P)**		
					**Researcher (R)**		
[19]	Decision making	26(26)C	Not stated	Selected clips only	P (clips by researcher)	‘Think aloud’ technique	Pre-consultation interview
		9GP		Transcripts from previous interview		Individual topic guides for interviews ‘designed to promote reflection’	Consultations
		9Pt^b^					Immediate post consultation interview
							VSR interview GP
							VSR interview Pt
[14]	Decision making	162(86)C	Immediately post	More than one video consultation	Video not stopped	Video shown first, semi structured interview following. Consultations selected for VSR chosen to reflect different discussions regarding smoking	VSR Interview GP
		39GP					(consultations analysed in other paper)
[18]	Decision making	72C	‘shortly after’	Video	P and R	Asked to stop video whenever wanted to comment generally or about preventative service plus semi structured interview	Pt VSR interview
		36Pt					GP VSR interview
		18GP					
[36]	Shared decision making	22(18)C	Within 2 weeks	video	P	P asked to stop when identified thoughts, feelings or behaviours associated with decision-making, followed by semi-structured interview	Pt VSR interview
		10GP					GP VSR interview
		18Pt					Consultations
[21]	Impact of computer on doctor patient relationship	39(39)C	1 week	Video	P and R	Interview guided by video analysis	Consultations
		12Pt					Pt VSR interview
		5GP					GP VSR interview
[39,40]	Impact of computer on doctor patient relationship	29C	Not stated	Video	Not stopped	Separate interview and video viewing. GP completed questionnaire when viewing the video	GP post consultation interviews
		6GP					GP questionnaire completed when watching video
							Consultation
							Observations at 4 sites [39]
							Pt interviews
[16,29]	Describe self-management interactions	86(40)C	1 week	video	P and R	Semi structured interview and prompts during playback	Patient post consultation interviews
	Impact of computer	11GP					6 VSR interviews (Nurses)
							Consultations (CA [16])
							GP VSR interview
[17,22-28]	Evaluation of SR method [22]	46C	About 1 week	Video, shown more than once	P	No interview. P asked to say what thinking. Neutral prompts if no response.	Pt VSR comments [17,22-24,26]
	Difficult consultations [24,25,28]	46Pt				GP asked to comment if unsure how to proceed	GP VSR comments [17,22,25-28]
	Doctor patient relationship [23,26,27] Health promotion [17]	12GP					Pt and GP questionnaire post viewing (effect of video on behaviour and satisfaction with consultation) [22]
							Consultation [17,23,25]
		(8C, 5GP, 8Pt)					
[30]	Doctor patient relationship	32C	Immediately	Video	P	Asked to say stop when they recalled thought or feeling	Satisfaction questionnaire
		16GP					(post consultation)
		32 Pt					GP VSR comments
							Pt VSR comments
[32]	Doctor-patient relationship	30C	Not stated	Video	P	P asked to comment on effective communication, things that were new, significant, unusual or important	Pt VSR comments and GP VSR comments edited in to original consultation tape for analysis
		15GP					
		30Pt					
[38]	Doctor patient relationship	1C	Not stated	Video	P	P asked to identify problems and concerns	Consultation
		1GP 1Pt					Medical record
							Pt VSR comments
							GP VSR comments
[15]	Communication	78(31)C	Not stated	Video	P and R	P asked to stop if any comment, particularly about HIV. R stopped tape after HIV discussion	Consultation
	Doctor patient relationship	26Pt				Semi structured interview after viewing	Pt VSR interview
	Difficult consultations	17GP					GP VSR interview
[34,35]	Communication	24C	Not stated	Video	P and R	R stopped for ‘key moments’, when interpreter did anything other than translate. Semi-structured interview	GP VSR interviews [34]
		24GP					Interpreters VSR interviews [35]
		22C					
		15 Inter-preters					
[20]	Cross cultural communication	25C	As soon as possible	Video	Video not stopped	Structured Interview post viewing	Pt VSR interview
		25P					(consultation analysed in other paper)
[33]	Cross cultural communication	U	P immediately	Video	P	Questioned first about recall, then asked to stop tape at any point	Pt VSR interview
		44pts	GP not stated				GP VSR interview
		UGP					Consultations
[13]	Cross cultural communication	24(24)C	Within 2 weeks	Video	P and R	R stopped for ‘key moments’ around cross cultural communication	Pt VSR interview
		12GP				Semi-structured interview	GP VSR interview
		24Pt					
[31]	Patients covert agenda	121C	Within 8 days	Video	P	3 phases: unprompted recall of consultation; asked to comment on any topic during video; then prompted by transcript of consultation	Pt VSR interview
		18Pt		Written transcript of consultation			
[37]	Clinician-patient relationship and team working	24Pt	One week	Video	P	Asked to stop tape and comment spontaneously	Pt VSR comments
		3GP^c^					GP VSR comments
							Other team members VSR comments

^a^Number of consultations collected (analysed) (C); Number of GPs undergoing VSR (GP); Number of patients undergoing VSR (Pt).

^b^Primary care data only, study included 14 other health professionals and 11 other patients.

^c^Unclear how many consultations as the 24 patients saw more than one member of the team.

Unfortunately, there is no empirical evidence from this review to comment on the importance of the timing of the VSR event or the strength of the stimulus, due to either a lack of reporting or lack of process evaluation. With regard timing of VSR, 10 papers did not report the length of time between video and VSR event. Of the other 18 studies, the VSR event occurred immediately post consultation in two, and up to two weeks later in the remainder. It was not possible to assess whether the studies with longer intervals had poorer recall. Bugge et al. [19] employed more than two post consultation interviews and for some participants, a further telephone interview at six months; it was not clear in this study how the additional post consultation reviews contributed to the results, or how recall differed in each review.

Three author groups enhanced the strength of the stimulus by either showing the video more than once, or by giving the participant a written transcript in addition to the video. Unfortunately, these studies did not evaluate to what extent the additional stimulus elicited additional information from participants.

A number of studies adopted participant-led low structure procedures where the participant was asked to comment on the video with no associated semi-structured interview, and neutral prompts only. As previously suggested, this method would be recommended for exploring decision making; however none of these studies were primarily concerned with decision making. Some studies did not report the nature of the prompts that were given to participants. Examples of prompts that were reported are listed in Table 10. A low structure procedure allows the participant to specify what is discussed but in some cases this method yielded little data. Arborelius et al. [22] stated that patients are less likely to comment spontaneously than doctors and Rosenburg et al. [13] and Epstein et al. [15] also reported low frequency of comments from patients. In some instances, the small amount of yielded data affected the robustness of the study conclusions, particularly if no additional data was analysed. In a study about the characteristics of a ‘human relationship’ with a doctor, analysis hinged on 21 of the original 227 patients’ spontaneous comments that related to this subject [23]. When doctors were asked to comment on the video with no specific line of inquiry, they usually focused on deficiencies in their behaviour; in one instance the conclusions of the study focused on doctor deficiencies as a result although the study question concerned GP experiences of the consultation [27].

**Table 10:** Examples of prompts given by researcher during VSR

Stop the tape when you felt uncertain as how to go on	[28]
Comment on anything new, unusual or different	[22]
What do you think when you look at the videotape?	[22]
Stop the tape when you identify thoughts feelings or behaviours associated with decision making	[36]
Stop the tape at moments you feel important or where you wish to comment, describe what you were thinking or feeling (Preceded with reminder of study focus - communication and cultural differences)	[13]
Tell me what was happening	[43]

Conversely, in the studies exploring decision making, there was limited acknowledgement of the possible influence of a semi-structured interview and researcher behaviour in altering participants’ accounts of consultation events. However, the use of semi-structured interviews generally elicited more information specific to the research question. Only one study did not use face to face VSR, but instead used a questionnaire to capture GPs’ thoughts during video playback in addition to a face to face interview (without VSR); again, the authors did not make clear in the results how the questionnaire results contributed to the findings of the study [39,40].

### What does VSR add? The contribution of VSR to findings

#### ***VSR to explore participants’ perceptions***

VSR was shown to have advantages over a non-stimulated interview approach in three studies with GPs. Firstly, in a study of discussion around smoking cessation, doctor participants showed great surprise at their actions on video; it was apparent from findings presented that the videos had uncovered aspects of behaviour that the GPs had previously not given any thought to, such as the impact of the computer on smoking cessation discussion [6,14]. GPs incorporated commentary on the patient’s nonverbal response to smoking cessation (viewed on video) to elaborate their accounts. Furthermore, the GPs in this study were asked about the *absence* of smoking related discussion and without VSR to cue the specific times when smoking could have been discussed, one can hypothesize that un-stimulated recall may not have been as effective. This work showed the importance of the context in which doctors practice in influencing smoking discussions, explaining why few doctors choose to discuss this issue with patients. Coleman et al. attributed the utility of the method to the subject of interest (smoking cessation) being mundane and therefore easily overlooked, and forgotten. In a similar vein, Blakeman et al. reported that VSR was useful for researching ‘taken for granted practice’. In their study regarding self-management, a GP expressed annoyance when watching himself weighing a patient revealing insights about the doctor’s perceptions of roles, an issue that one can speculate may have been overlooked in a non VSR interview [16]. The third example concerns GPs’ reactions to their discussions around HIV risk [15]. The GPs in this study were ‘generally surprised’ at their actions and offered unexpected insight into communication barriers, such as the importance of the lack of a simple opening statement in starting HIV risk discussion.

Of the other studies researching patient experiences the added value of VSR was unclear [20,24,31,37]. There were no reports of patients showing surprise at the video findings, as has been noted in several VSR interviews with GPs [6,15,21]. One interpretation may be that VSR is more useful for enhancing reflection in clinicians; however, the studies with patients had a number of methodological limitations. In general, the lack of detail around methods was accompanied with insufficient detail in results to judge the added value of VSR.

#### ***VSR to explore non-spoken behaviours***

In two studies, non-verbal events were the focus of the research question and the VSR. Bugge et al. [19] explored the significance of non-disclosure of information during decision making. In this study the value of VSR was evident; clinicians reported information they typically sought in certain decision making situations, but the video consultations revealed the absence of the reported behaviour. During the VSR interviews the authors were able to unpick the reasons for non-disclosure including assumptions about patient preferences and uncertainty about treatment effectiveness. As clinicians were clearly not aware of some episodes of non-disclosure prior to viewing, a non-stimulated interview could not have reached the same findings. This study also gives further weight to the suggestion that VSR may be particularly useful for doctors.

Henry et al. [18] identified how tacit clues, including non-verbal behaviours, subconsciously inform clinical judgements. In this study, patients were found to be very attuned to doctor body language and doctors often unaware or unable to articulate rationale behind their judgements; however, doctors were found to have a varying sensitivity to tacit clues. Both of these studies have useful implications for our understanding of doctor patient communication and necessitated a VSR approach due to the specific nonverbal or nondisclosure event in the consultation that needed further elucidation.

#### ***VSR in conjunction with consultation analysis***

In this review, the included studies varied in the extent to which different sources of data contributed to the overall analysis, as detailed in Table 9. In the studies where the consultation was analysed alongside the VSR interviews, a number of different methods of analysis were used. Analysis was conducted both ‘across cases’, and ‘within cases’. In across case analysis, VSR interviews were analysed as a whole with no comparison to the relating consultation; in within case analysis, the consultation and VSR transcripts pertaining to one consultation were analysed together.

In the studies using within case analysis, the added value of using VSR was clearly evident. The use of VSR was particularly illuminating in a study exploring shared decision making and the experience of partnership. By comparing and contrasting physician and patient views on episodes of decision making, Saba et al. have been able to shed light on previous work that has identified discordance between satisfaction and shared decision making in consultations [36]. This study has demonstrated that shared decision making could occur in the presence of mistrust and frustration, and they conclude that both good communication and relationship dynamics are necessary for shared decision making. A further example of the strength of the within case analysis approach comes from Rosenburg at al’s study of intra-cultural encounters [13]. The detailed descriptions in the paper of consultation excerpts alongside patient and doctor responses during interview enabled the authors to draw novel insights about areas for improvement in intracultural encounters, again with important educational implications.

The use of VSR to study specific instances of sensitive talk around HIV risk [15] was also very successful in identifying the successful elements of HIV risk discussion, with educational implications. Although the VSR component seemed to contribute a small amount to the study findings (compared to consultation analysis), the GP interviews did appear to be useful in eliciting the nature of barriers to effective discussion. In this example, the research participant almost becomes researcher, aiding the interpretation of findings.

In studies that analysed consultations in a silo independent of VSR findings (across case analysis), the analysis was felt to be lacking in depth and rigour with missed opportunities for insight from the data [21,26].

## Conclusions

This review highlights that VSR is particularly useful for the study of specific consultation events when analysis adopts both a within and across case approach. For enhancing participant recall, VSR may be particularly relevant for topics which are routine and easily overlooked, for interviewing doctors and for exploring non-spoken and non-verbal behaviour. The method may be particularly useful for exploring clinicians’ perceptions, as differences in rhetoric and behaviour can be explored; the use of interviews alone to research doctor perceptions has been criticised [44,45] and VSR may provide a useful alternative. Blakeman et al. [16] state that the method helps to explore interactions that may have remained unremarkable to both participant and researcher, particularly where the researcher has the same professional background as the participant (‘shared conceptual blindness’).

In reviewing study quality, frequently there was insufficient reporting of methods to properly evaluate this; one contributory factor to this may be that many journals’ word limits may not facilitate proper reporting of complex methodology. Ensuring the technique of VSR, the study sampling and the choice of data sources align to the research question have emerged as particularly important elements in the quality of these studies. VSR studies may generate a lot of data, and care needs to be taken to ensure data collected are relevant to the research question, and represented in the study findings. Studies identified in this review have generally not used opportunities to evaluate their methods e.g. by reporting how un-stimulated recall compared to recall, or how different aspects of data contributed to findings.

As stated in the introduction, there is concern, particularly in the psychology and sociology literature about the method of VSR producing ‘a second-order reconstituted account’ [46], influenced by the degree of researcher ‘interference’ in the process of VSR. Few authors commented on this limitation, with some [15] stating the counter argument, that using participants as experts to interpret their own behaviour yielded unexpected issues. To some extent the argument here will be influenced by a researcher’s theoretical and epistemological viewpoint; a post-positivist approach would align with the need to maximise validity and reduce researcher interference, whereby an interpretivist approach would sit more comfortably with the need to respect the differences between viewpoints and make sense of findings using the meanings derived from the ‘actors’ within the consultation. In the papers included, researchers did not make their viewpoint explicit. However, the majority of studies did aim to elucidate participant experience in some way, and as such vigorous attention to validity of recall may be less important than research in other disciplines where the concern is to accurately reflect cognitive processes.

In this review, studies which have tried to reduce researcher interference, for example by using only neutral prompts during VSR, have often resulted in small amounts of data, much of which was unrelated to the research question. This may have been due to lack of participant or researcher training in the method. The findings of this review suggest that although the limitations of moderate to high structure reviews/ post consultation interviews should be acknowledged, that these methods usually resulted in richer data related to the research question than low structure, participant-led approaches. Prompts given by researchers during playback may still remain ‘neutral’ while providing a context e.g. study aim or orientation for the participant to comment.

VSR is an intrusive methodology and it is likely that ethical issues arise during the conduct of these studies, such as patient distress during video review. Guillemin and Gillam refer to this as ‘ethics in practice’ as opposed to ‘procedural ethics’, concerned with consent processes and formal approval [47]. No study referred to any ethical issues arising during data collection. Related to this is the issue of acceptability, and how participants react to VSR, which remains unknown.

Lomax [46] argues a reflexive stance is essential when collecting video data as the entire research process has a distorting effect on ‘real life’. Increased reporting of the ethical issues ‘in practice’ and the influence of the researcher on the process and would increase the quality of reporting of these studies. These issues are common to other qualitative research [48], although particularly relevant to VSR, as evidenced by the distress during VSR described in one study [15].

This review was conducted with a systematic search. Searching all papers containing reference to video for evidence of VSR, instead of restricting the search by identified terms for VSR, has identified more studies than a previous literature review [5], which also did not quality appraise identified studies. A strength of this review is the use of quality assessment, using the CASP tool [10] to both inform results and underpin conclusions. Furthermore, the use of the classification described by Gass and Mackey as a theoretical framework to inform analysis has resulted in practical conclusions that will hopefully assist researchers considering the use of the method. No study was excluded based on methodological quality and the heterogeneity of studies may limit the robustness of the synthesis. The most striking difference was in the design and reporting of participant consent in older studies, possibly conducted in an era where the use of video was not as widespread as it is today.

In summary, this systematic review furthers understanding of both the role of VSR in understanding the consultation and the methodological strengths and weaknesses of this approach. Future researchers using the method may consider factoring in process evaluation to gain further understanding of how VSR contributes to recall, the acceptability to participants and how changes to methodology influence findings.

## Abbreviations

CASP: Critical appraisal skills programme; GP: General practitioner; SR: Stimulated recall; VSR: Video stimulated recall.

## Competing interests

The authors declare that they have no competing interests.

## Authors’ contributions

ZP conceived the study, designed the protocol, conducted literature searches, selected papers for inclusion, undertook data extraction, quality assessment, conducted the narrative synthesis and drafted the manuscript. GMcH selected papers for inclusion, undertook data extraction and quality assessment, and contributed to narrative synthesis. ABH undertook data extraction and quality assessment, and contributed to narrative synthesis. All authors read and approved the final manuscript.

## Authors’ information

ZP is a Clinical Lecturer and Honorary Consultant Rheumatologist and conducted this systematic review as part of a PhD using VSR methodology to explore the osteoarthritis consultation in primary care. This is part of a larger programme of work aiming to enhance the care of osteoarthritis in primary care; GMcH is a Senior Lecturer with experience in mixed methods research who is working on this research programme. ABH is a Professor of Medical Education and Consultant Rheumatologist and PhD supervisor to ZP.

## Pre-publication history

The pre-publication history for this paper can be accessed here:

http://www.biomedcentral.com/1471-2288/14/101/prepub

## Supplementary Material

Additional file 1 **Example Search in Medline.** Full search history conducted in Medline database. Click here for file

Additional file 2 **Data extraction form.** Full form as used by authors including data extraction and quality appraisal components. Click here for file
