# Supplementary material for: Getting under the skin of the primary care consultation using video stimulated recall: a systematic review
Source: BMC Med Res Methodol. 2014 Aug 30;14:101. doi: 10.1186/1471-2288-14-101 (PMC4154900; doi:10.1186/1471-2288-14-101)
Supplement: Additional file 1 — Example Search in Medline. Full search history conducted in Medline database. [file 1471-2288-14-101-S1.docx]

# Additional File 1: Example search in Medline

1. consult*.ti,ab.

2. "Referral and Consultation"/

3. Communication/

4. Physician-Patient Relations/

5. (doctor adj5 patient).ti,ab.

6. (GP adj5 patient).ti,ab.

7. (physician adj5 patient).ti,ab.

8. (clinician adj5 patient).ti,ab.

9. ("general practitioner" adj5 patient).ti,ab.

10. (talk* or rapport* or relation*).ti,ab.

11. 5 or 6 or 7 or 8 or 9

12. 10 and 11

13. communicat*.ti,ab.

14. 1 or 2 or 3 or 4 or 12 or 13

15. family practice.ti,ab.

16. Family Practice/

17. physicians, family/ or physicians, primary care/

18. Primary Health Care/

19. GP.ti,ab.

20. "family medicine".ti,ab.

21. "family doctor*".ti,ab.

22. "general practi*".ti,ab.

23. "family physician*".ti,ab.

24. 15 or 16 or 17 or 18 or 19 or 20 or 21 or 22 or 23

25. videotape recording/ or video recording/ or videodisc recording/

26. video*.af.

27. (digital adj2 record*).ti,ab.

28. (disc adj2 record*).ti,ab.

29. Tape Recording/

30. film*.ti,ab.

31. recording*.ti,ab.

32. Interview/

33. "Attitude of Health Personnel"/ or Attitude to Health/

34. qualitative research/

35. qualitativ*.ti,ab.

36. interview*.ti,ab.

37. experience*.ti,ab.

38. finding*.ti,ab.

39. theme*.ti,ab.

40. account*.ti,ab.

41. 32 or 33 or 34 or 35 or 36 or 37 or 38 or 39 or 40

42. 25 or 26 or 27 or 28 or 29 or 30 or 31

43. 14 and 24 and 41 and 42
