# Supplementary material for: Getting under the skin of the primary care consultation using video stimulated recall: a systematic review
Source: BMC Med Res Methodol. 2014 Aug 30;14:101. doi: 10.1186/1471-2288-14-101 (PMC4154900; doi:10.1186/1471-2288-14-101)
Supplement: Additional file 2 — Data extraction form. Full form as used by authors including data extraction and quality appraisal components. [file 1471-2288-14-101-S2.docx]

# Additional File 2: Form for data extraction for systematic review

**Reference information**

1. Reviewer:
2. Date:
3. Paper 1^st^ author:
4. Title:
5. More than one paper for this data set? [drop down] Yes/ no/ don’t know
6. If yes, Index paper where data collection fully described (if applicable) [free text]
7. Include: [drop down] yes; no; don’t know
8. Reason for exclusion: [drop down] educational research; standardised patients or actors; non English; not primary care; not GPs (other healthcare professional); not observational study; video not shown to research participants; other
9. Study classification: [drop down] Decision making; Doctor-patient relationship; condition specific information; other [free text]

Study information

1. What is the research question? [free text]
2. How were consultations selected? Tick all that apply

[drop down] Screened; consecutive; disease specific; GP provided tapes; researcher consented; other [free text]

1. Who were the population of interest? [free text]
2. How many consultations were videoed? [free text]
3. How many consultations were analysed? [free text]
4. What are the main findings? [free text]
5. What methods have been used for analysis of consultations? [drop down] Not analysed; Conversation analysis; discourse analysis; qualitative; checklist – bespoke; checklist – RIAS; checklist – other; timing of consultation or other timings (give details) [free text}; other [free text]
6. How has the visual data been analysed? [free text]
7. What other data collection was performed? Tick all that apply and give details: [drop down] Patient questionnaire pre-consultation; Patient questionnaire post-consultation; GP questionnaire pre-consultation; GP questionnaire post-consultation; Patient interview post- consultation; GP interview post- consultation; Patient interview post- consultation with video; GP interview post- consultation with video; focus groups post consultation; other [free text]
8. How many interviews using video were conducted? With GPs [free text], With patients [free text]
9. How were the videotapes selected? [drop down] GP chose; researcher chose
10. What format did the interview take? Tick all that apply and give details [ drop down] video shown in entirely first; video shown in clips (researcher paused); video shown in clips (participant paused); unstructured interview; semi structured interview; other [free text]
11. Has the researcher commented on acceptability to participants of viewing video in interview setting? [free text]
12. What are the authors’ main conclusions? [free text]
13. What are the reviewer’s main conclusions? Include comments on the value of the research [free text]
14. Did each component (interview vs video) contribute to the findings?
15. To what extent did the VSR interview add to the research findings ?

Quality assessment

1. Was the research design appropriate to address the aims of the research? [free text]
2. Was the recruitment strategy appropriate to the aims of the research? [free text]
3. Has the data collection been clearly described? [free text]
4. Was the data collected in an appropriate way to address the research question? [free text]
5. Have ethical issues been taken into consideration? [free text]
6. Has the relationship between researcher and participants been considered? [free text]
7. Has external validity been commented on?
   1. Characteristics of consenting patients [free text]
   2. Characteristics of consenting GPs [free text]
8. Has internal validity been commented on?
   1. Effect of videoing on patients’ behaviour [free text]
   2. Effect of videoing on GPs’ behaviour [free text]
9. Was the data analysis sufficiently rigorous? [free text]
10. Is there a clear statement of findings? [free text]
